# Supplementary material for: Human cell transformation by combined lineage conversion and oncogene expression
Source: Oncogene. 2021 Jul 23;40(36):5533–47. doi: 10.1038/s41388-021-01940-0 (PMC8429043; doi:10.1038/s41388-021-01940-0)
Supplement: Supplementary file 1 — Supplementary Information [file 41388_2021_1940_MOESM1_ESM.pdf]

Supplementary Information for

Human cell transformation by combined lineage conversion and oncogene expression

Biswajyoti Sahu[1,2,3,4], Päivi Pihlajamaa[1,3], Kaiyang Zhang[5], Kimmo Palin[1,4,6], Saija Ahonen[1], Alejandra Cervera[5,7], Ari Ristimäki[1,2,8], Lauri A. Aaltonen[1,4,6], Sampsa Hautaniemi[5] and Jussi Taipale[1,3,9]*

Supplementary Information includes:

Supplementary Figures S1 to S9

Supplementary Tables S1 to S4

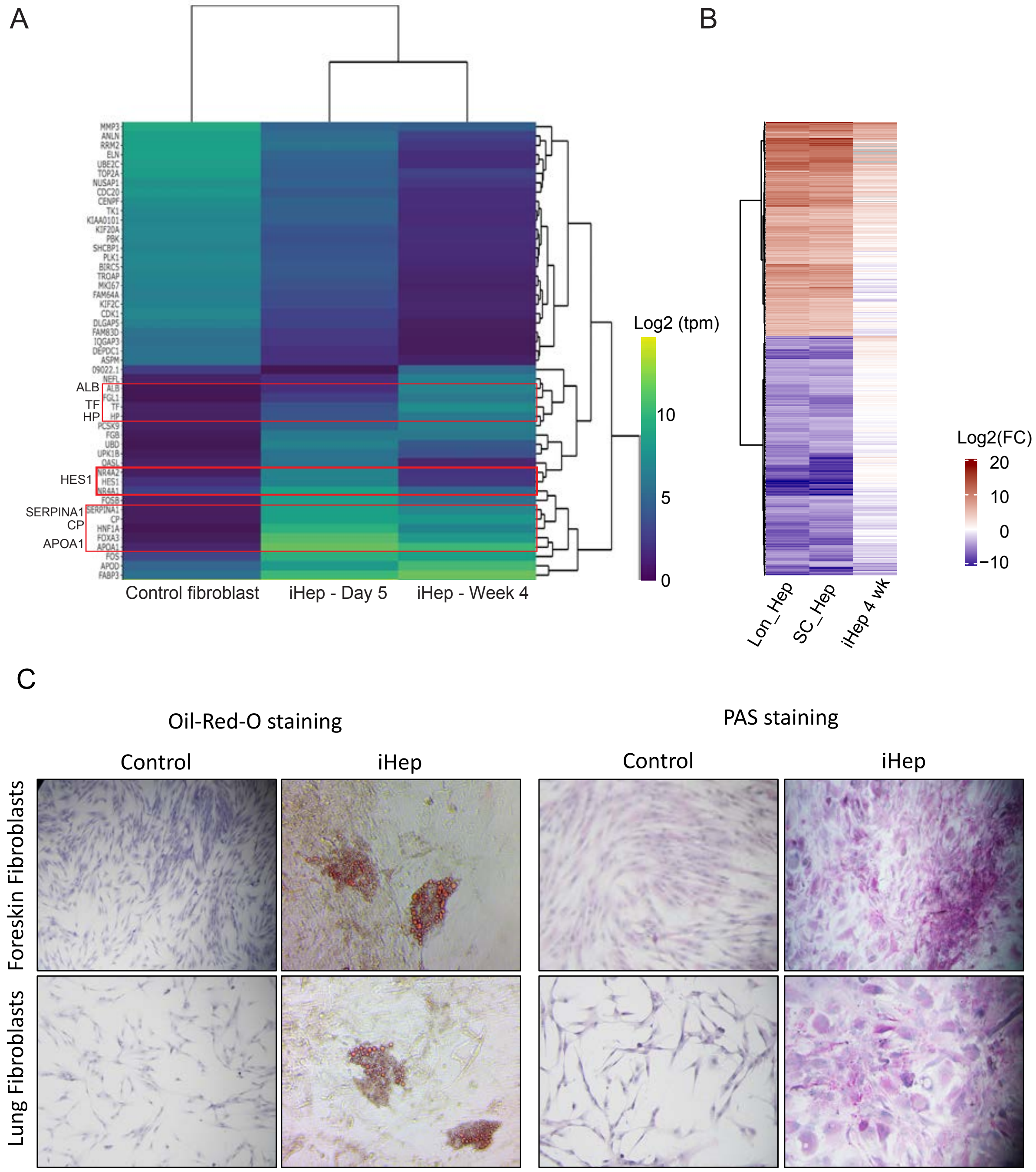

Supplementary Fig S1. Characteristics of induced hepatocytes. A Heatmap showing the gene expression changes measured by RNA-seq during the conversion of human fibroblasts to induced hepatocytes (iHeps) at early time-point (5 days post-transduction) and at 4 weeks of iHep differentiation with hepatocyte marker genes and Notch signaling pathway genes highlighted in red. Log_2(transcripts per million, TPM) values for collated triplicates are shown. **B** Heatmap showing log_2(fold change, FC) values from RNA-seq analysis for two primary human hepatocytes, Lon_Hep (Lonza) and SC_Hep (ScienCell), and iHeps at 4 weeks of transdifferentiation against control fibroblasts (mean, n=3). Genes differentially expressed in both primary hepatocyte samples against control fibroblasts are shown for all conditions (see **Materials and methods** for details). **C** Oil-Red-O staining showing lipid accumulation and Periodic Acid-Schiff (PAS) staining showing glycogen storage for iHeps at 6 weeks post-transduction along with respective control fibroblasts.

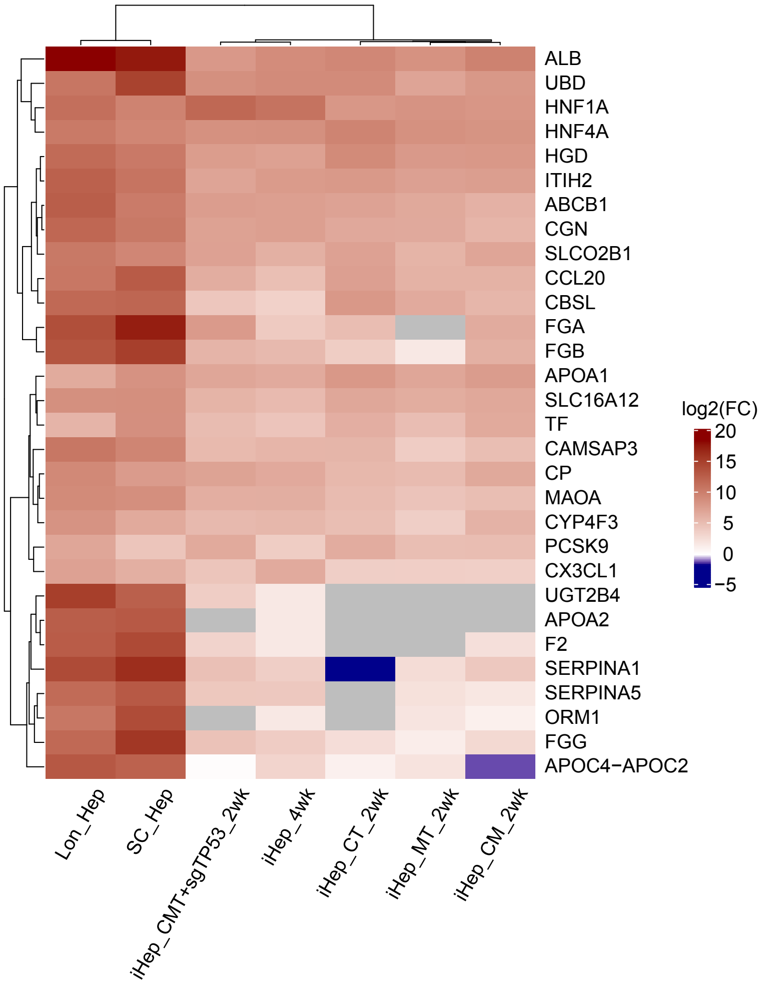

Supplementary Fig. S2. Comparison of oncogene-transduced iHeps to primary hepatocytes. Heatmap showing log_2(fold change, FC) values from RNA-seq analysis against control fibroblasts for two primary human hepatocytes, Lon_Hep (Lonza) and SC_Hep (ScienCell), iHeps at 4 weeks of transdifferentiation, and iHeps transduced with CMT+sg*TP53* (mean, n=3), as well as for iHeps transduced with combinations of two oncogenes (CT, CTNNB1+TERT; MT, MYC+TERT; CM, CTNNB1+MYC; mean, n=2) two weeks after oncogene transduction. Top 30 up-regulated genes (from **Fig. S1B**) are shown.

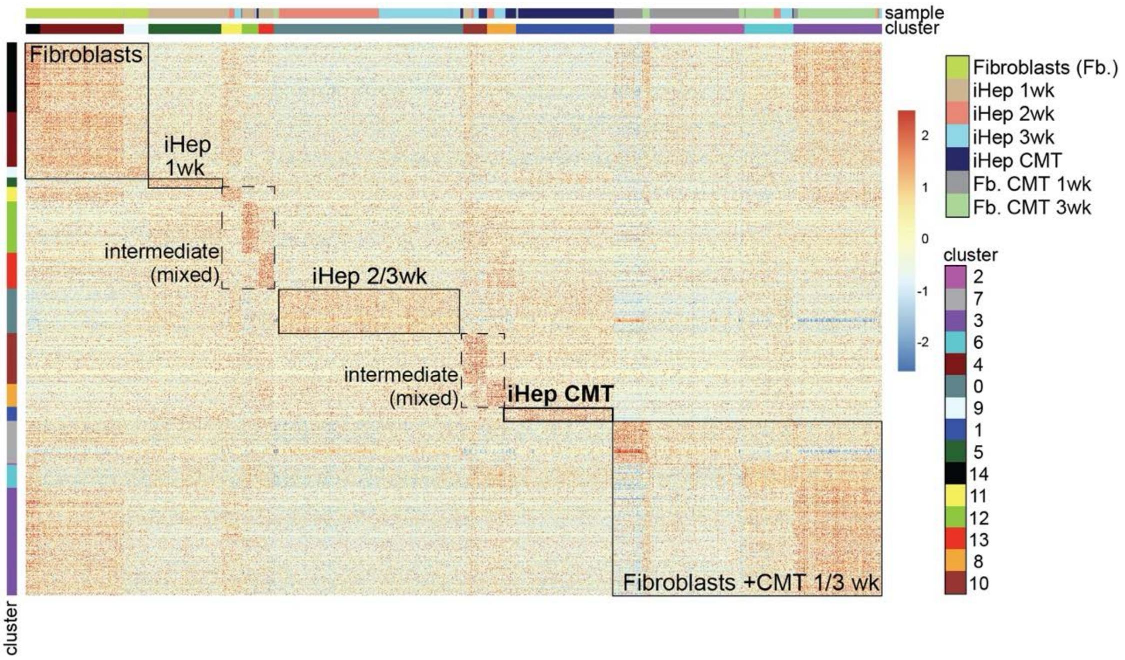

Supplementary Fig S3. Marker genes from scRNA-seq analysis. Clustered heatmap showing the expression levels of cluster-specific marker genes (the expression of a gene in a particular cell relative to the average expression of that gene across all cells), with Bonferroni corrected p-value < 0.01 and average $\log_2$ fold change > 0.2 or < -0.2, from single cell RNA-seq analysis. Color code illustrating sample and cluster identities correspond to the colors in **Fig 4A and B**.

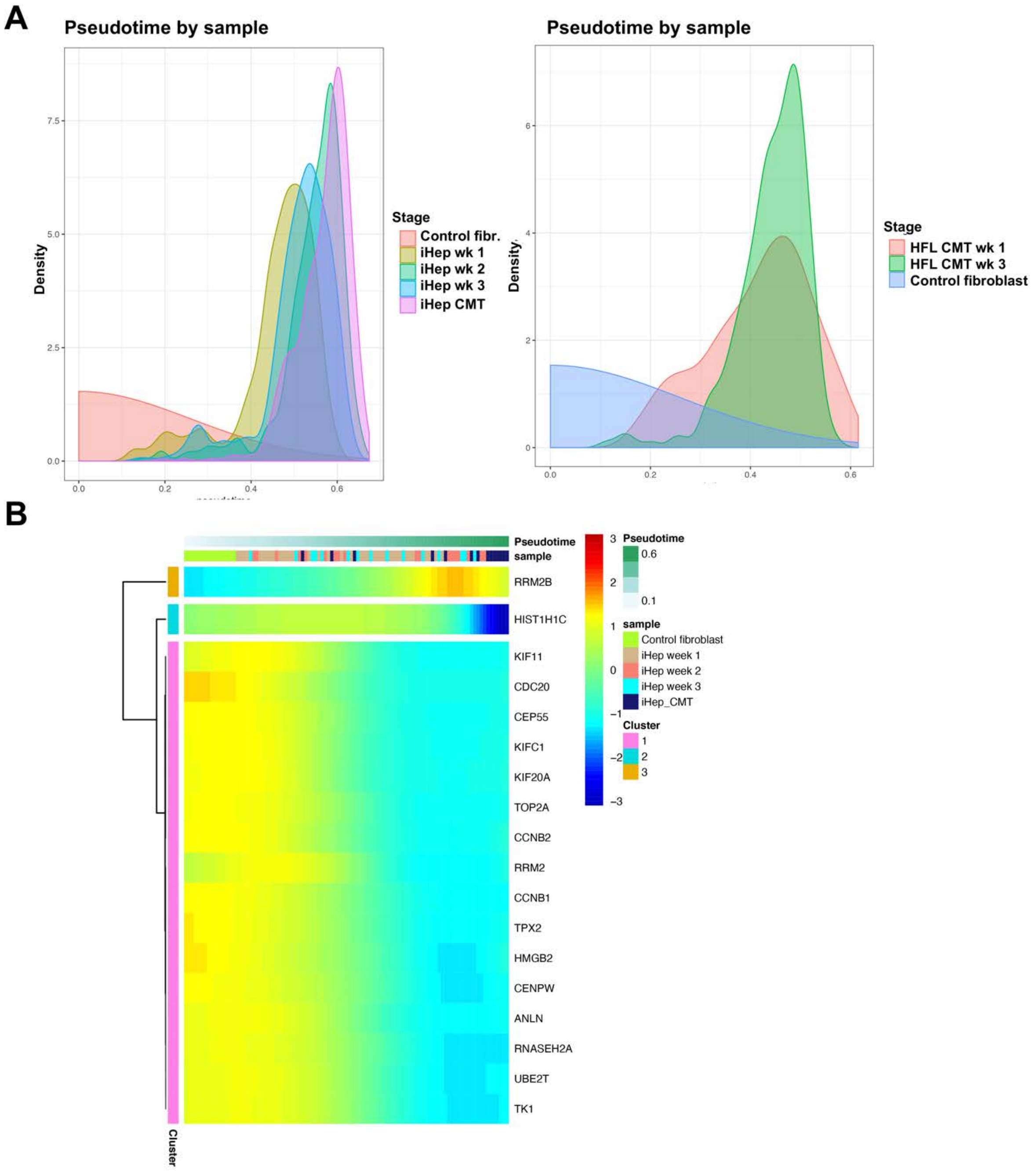

Supplementary Fig S4. Gene expression dynamics during iHep differentiation and transformation. A Distribution of different scRNA-seq samples along the pseudotime. **B** Heatmap showing the expression dynamics for senescence marker genes [from Marthandan*, et al.,* (2016) as cited in the main text] along the pseudotime of iHep differentiation and transformation in the scRNA-seq data from control fibroblasts, iHeps at one–three weeks after iHep induction, and CMT-iHeps two weeks after oncogenes (the expression of a gene in a particular cell relative to the average expression of that gene across all cells is shown). Color codes illustrating sample identities correspond to the colors in **Fig. 4A**.

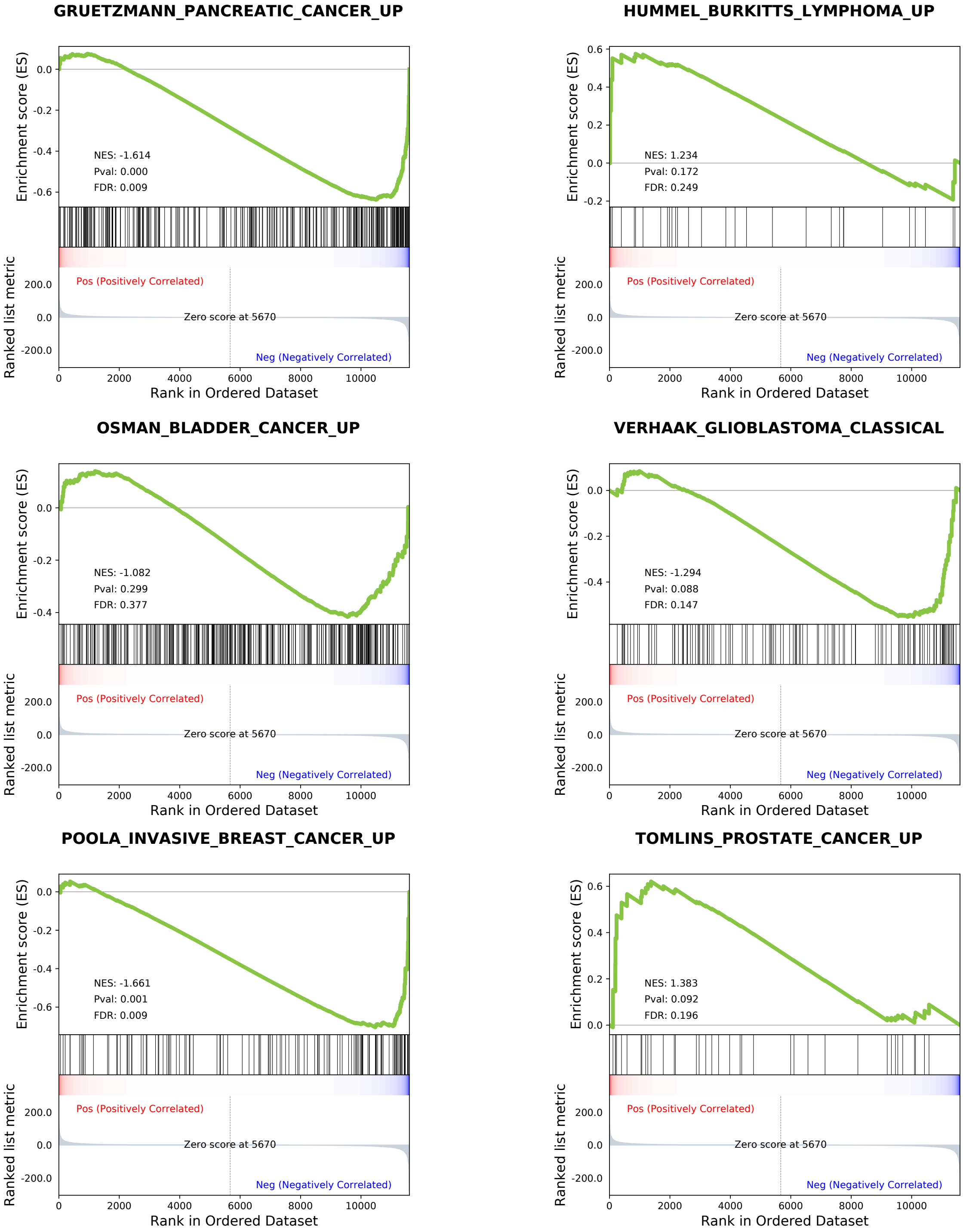

Supplementary Fig S5. **Correlation of gene expression signature of transformed iHeps to cancer types other than liver cancer.** Gene set enrichment analysis for transformed iHep-CMT+sg*TP53* cells at p20 compared to control fibroblasts against cancer signatures from molecular signatures database (MSigDB).

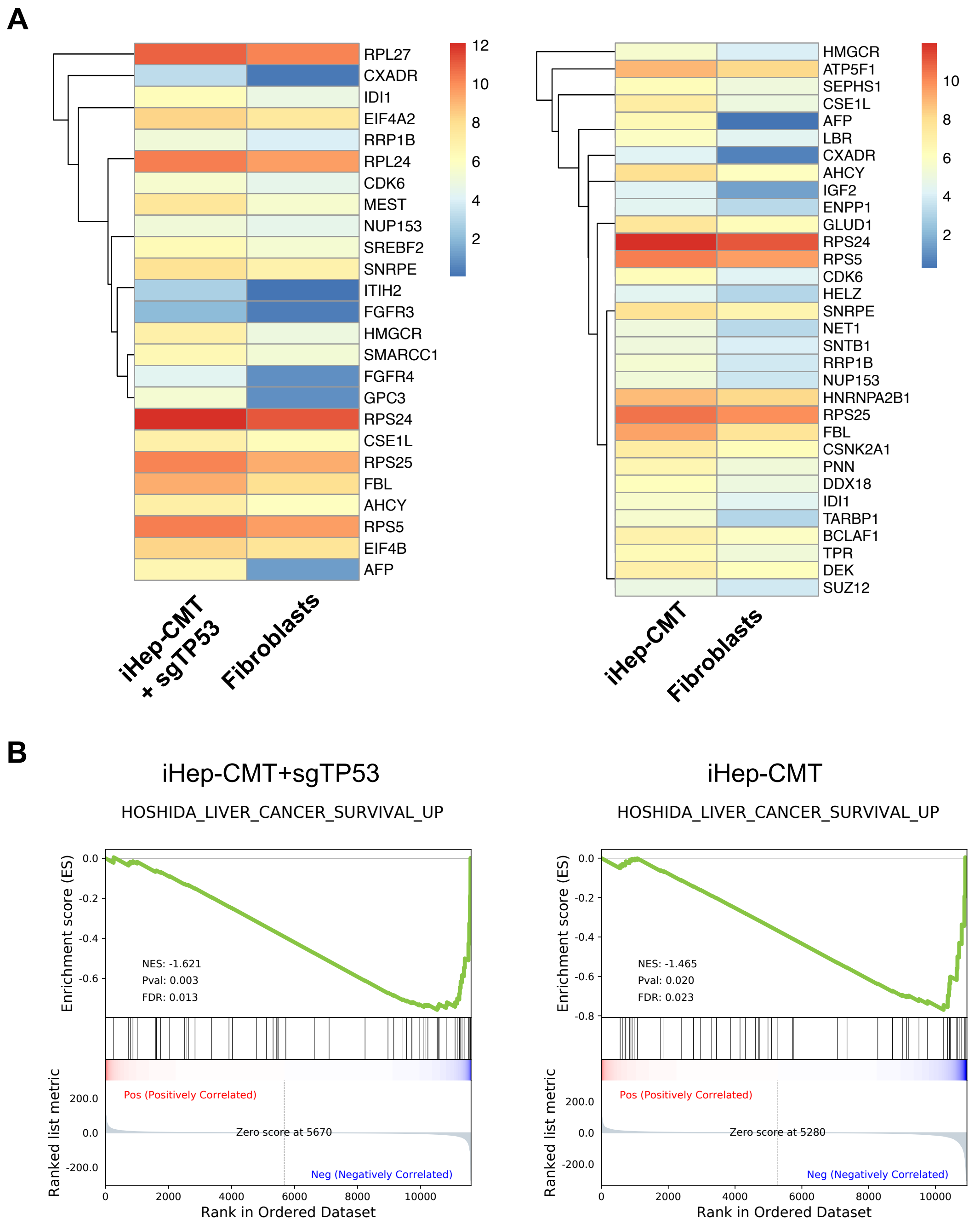

Supplementary Fig S6. Liver cancer signatures analyzed in transformed iHeps. A Expression of the leading-edge subsets of genes from gene set enrichment analysis against Subclass 2 liver cancer signature [from Hoshida *et al.,* (2009) as cited in the main text] in CMT-iHeps and CMT+sg*TP53*-iHeps compared to control fibroblasts (scale bar showing mean expression values). **B** Gene set enrichment analysis (GSEA) results for liver cancer survival genes [from Hoshida *et a*l., (2009) as cited in the main text] in transformed iHeps compared to control fibroblasts.

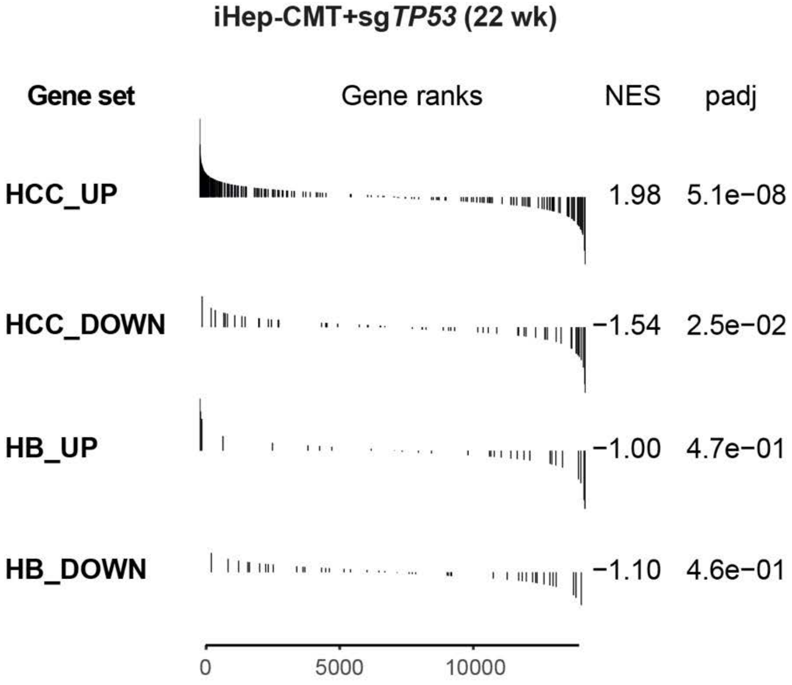

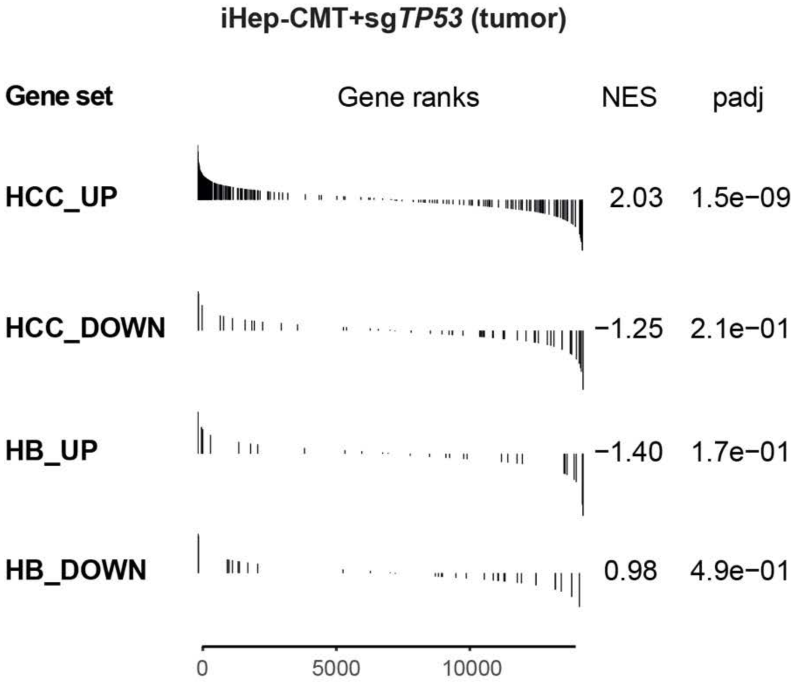

Supplementary Fig S7. Hepatocellular carcinoma (HCC) and hepatoblastoma (HB) signatures analyzed in transformed iHeps. Gene set enrichment analysis of differentially expressed genes analyzed from RNA-seq data in transformed iHep-CMT+sg*TP53* cells at p20 (~22 weeks after oncogene transduction; top) and xenograft tumor generated from them (bottom) vs. iHep control against HCC- and HB-signatures generated from previously published gene expression data sets for human HCC and HB tumors compared to normal liver (see **Materials and methods** for details). Note the positive normalized enrichment score (NES) significantly associated to HCC_UP signature but not to HB_UP signature, and negative NES associated to HCC_DOWN signature.

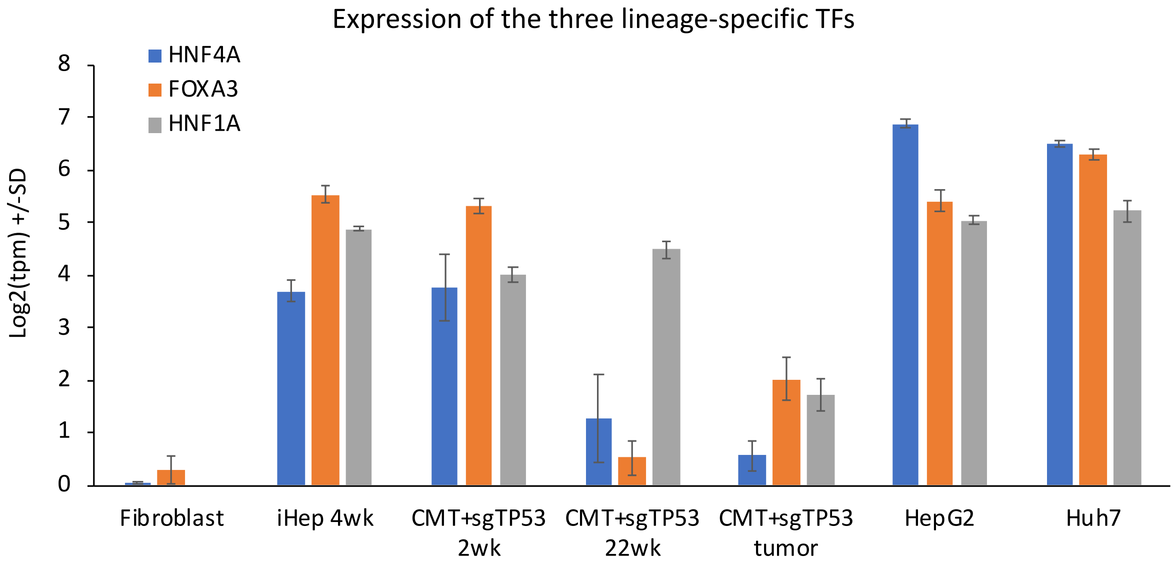

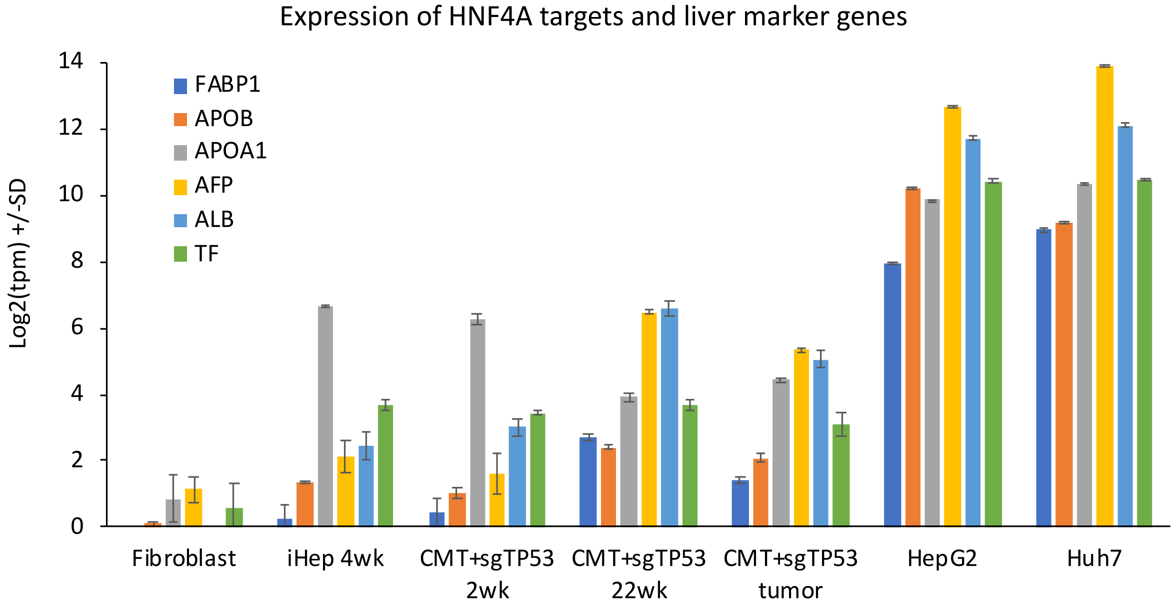

Supplementary Fig S8. **Expression of TFs and marker genes during transdifferentiation and transformation**. Gene expression levels measured by RNA-seq from human liver cancer cells lines (HepG2 and HuH7) and during the conversion of human fibroblasts to induced hepatocytes (at four weeks of differentiation) and to highly proliferative tumorigenic cells (CMT+sg*TP53* cells shown at two and 22 weeks after oncogene transduction, as well as RNA extracted from the CMT+sg*TP53* tumor). Mean from three biological replicates with standard deviation for log_2[transcripts per million (TPM)] is shown (total expression values combined from endogenous and lentiviral transcripts).

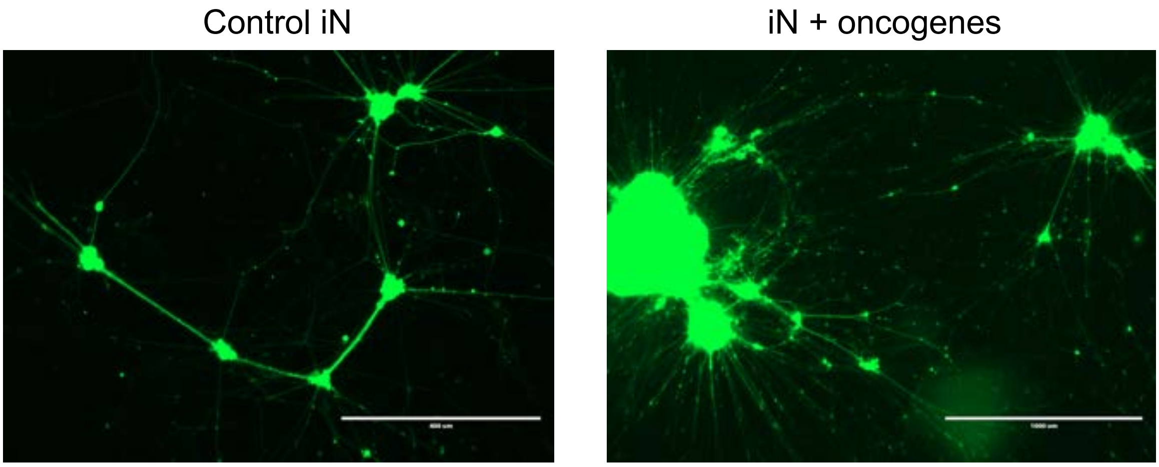

Supplementary Fig S9. Induced neurons reprogrammed from human fibroblasts using defined TFs. Fluorescent microscope images (40x) of induced neurons with and without transduction of medulloblastoma-specific oncogenes (at three weeks of neuronal differentiation) visualized using EGFP at ten weeks after neuronal conversion.

Supplementary Table S1. Genomic insertions of lentiviral constructs. Number and genomic loci for insertions of lentiviral expression constructs that were used for ectopic expression of lineage-specific TFs and oncogenes, detected in the sequencing reads from the NaNoMe-seq data from CMT+sg*TP53* cells at p20, 22 weeks after oncogene transduction.

A. Total number of insertions for each detected construct

Construct	Number of insertions
HNF1A	1
FOXA3	0
HNF4A	0
TERT	8
MYC	12
CTNNB1	0

B. Genomic coordinates for the insertions supported by more than one Nanopore read

Insertion locus			Lentiviral expression construct	Unique reads
ChrX	138839044	138839050	TERT	8
Chr1	27684639	27684641	Cas9	10
Chr1	43317914	43317916	TERT	18
Chr1	150921674	150921676	Cas9	9
Chr1	151770393	151770399	MYC	16
Chr1	226341541	226341544	TERT	12
Chr4	2867705	2867709	Cas9	9
Chr6	31611488	31611491	MYC	22
Chr6	32925912	32925917	TERT	17
Chr6	33227277	33227279	MYC	11
Chr6	74230721	74230727	Cas9	23
Chr6	109580023	109580027	HNF1A	21
Chr8	6384753	6384754	MYC	14
Chr9	32980138	32980141	MYC	17
Chr9	33904383	33904384	TERT	14
Chr9	130408513	130408518	MYC	9
Chr9	139308028	139308032	MYC	23
Chr10	17121499	17121510	Cas9	7
Chr12	7041910	7041912	MYC	19
Chr12	56691765	56691769	MYC	10
Chr15	43116574	43116580	Cas9	6
Chr16	360600	360606	TERT	4
Chr16	1887275	1887282	MYC	9
Chr17	27116562	27116564	MYC	20
Chr17	31064738	31064743	Cas9	8
Chr17	38564351	38564353	TERT	11
Chr17	57883662	57883668	Cas9	9
Chr19	46123309	46123313	MYC	10
Chr22	51029725	51029726	TERT	14

Supplementary Table S2. Examples of model systems for studying cell fate and tumorigenesis. See full citations in the list of references for the main text.

**Model system**	**Reference**	**Approach**	**Key features**
Mogrify	Rackham *et al*., (2016)	A predictive computational framework for identifying factors for direct cell fate conversion	A systematic approach that aids in the development of transdifferentiation protocols, experimental validation still needed for each case
Human cancer-derived organoids	Broutier *et al*., (2017)	Primary liver cancer-derived organoid cultures that recapitulate features of different liver cancer types	Enables drug testing and molecular analyses in a patient-specific manner, but each organoid culture represents an established tumor
Cancer-like cells generated from human primary cells	Park *et al*., (2018)	Oncogenic drivers introduced to normal prostate and lung epithelial cells generated small cell prostate cancer and small cell lung cancer cells	Enables studying defined oncogenic factors but limited by the availability of suitable primary cells and generation of neuroendocrine tumors instead of epithelial ones
Cancer-like cells generated from human embryonic stem cells	Chen *et al*., (2019)	Generation of pulmonary neuroendocrine cells and small cell lung cancer-like tumors from human embryonic stem cells (ESC)	Dynamic approach for studying oncogenic factors, but intermediate stage in the assay is already tumorigenic in mice (i.e., teratomas from ESCs)
Transdifferentiation–transformation from human fibroblasts using defined factors	This manuscript	Lineage-specific TFs are used for cell fate conversion and cancer-specific oncogenes for cellular transformation to generate liver cancer cells	Dynamic molecular model that enables studying the contribution of each factor of interest in a systematic manner and recapitulating early stages of tumorigenesis<br><br>Of note, using this model, the role of defined tissue-specific transcription factors identified through Mogrify can be investigated together with the major human oncogenic drivers identified by cancer genetic studies, offering a novel platform for studying lineage-specificity of human cancers (see Haigis *et al*., 2019).

Supplementary Table S3. Sequences of the primers used for qRT-PCR.

**qRT-PCR primer name**	**Primer sequence (5’ → 3’)**
GAPDH+	GGCCTCCAAGGAGTAAGACC
GAPDH-	AGGGGAGATTCAGTGTGGTG
ALB+	GGATGAAGGGAAGGCTTCGT
ALB-	GAAATCTCTGGCTCAGGCGA
TF+	GGCCACTAAGTGCCAGAGTT
TF-	ATCCAGTGTCACAGCATCCG
SERPINA1+	CTGTCTCCTCAGCTTCAGGC
SERPINA1-	CACGAGACAGAAGACGGCAT

Supplementary Table S4. Codon-optimized sequences used for lentiviral expression of *HNF4A*, *TERT*, and *CTNNB1* ^{T41A}

Gene name	Coding sequence (5’ → 3’)
HNF4A	ATGAGACTGAGCAAGACCCTGGTGGACATGGATATGGCCGACTACTCCGCAGCACTGGACCCCGCTTATACTACT<br>CTGGAATTTGAGAACGTGCAGGTCCTGACCATGGGGAATGATACATCCCCATCTGAGGGAACAAACCTGAATGCA<br>CCAAACTCCCTGGGCGTGTCTGCCCTGTGCGCTATCTGTGGGGATCGGGCAACTGGGAAGCACTACGGAGCCAGC<br>TCCTGCGACGGGTGTAAGGGATTCTTTCGGAGATCTGTGAGAAAAAACCATATGTACAGTTGCAGGTTCTCACGC<br>CAGTGCGTGGTCGACAAGGATAAACGCAATCAGTGCCGATATTGTCGGCTGAAGAAATGCTTTCGAGCCGGCATG<br>AAGAAAGAGGCTGTGCAGAATGAACGAGATCGGATTTCCACCAGGCGCTCTAGTTATGAAGACTCAAGCCTGCCT<br>TCAATTAACGCACTGCTGCAGGCTGAGGTGCTGAGCCGGCAGATCACATCTCCAGTCAGTGGCATTAATGGGGAT<br>ATCAGAGCCAAGAAAATCGCATCTATTGCCGACGTGTGCGAGAGTATGAAGGAACAGCTGCTGGTGCTGGTCGAA<br>TGGGCTAAATACATCCCTGCATTCTGTGAGCTGCCACTGGACGATCAGGTGGCTCTGCTGAGGGCACACGCCGGA<br>GAGCATCTGCTGCTGGGCGCAACCAAGCGCAGCATGGTGTTTAAAGATGTCCTGCTGCTGGGCAACGACTATATT<br>GTGCCTAGGCACTGTCCAGAGCTGGCCGAAATGTCAAGAGTGAGCATCAGGATTCTGGATGAGCTGGTCCTGCCA<br>TTCCAGGAACTGCAGATCGACGATAATGAGTACGCATATCTGAAGGCCATCATTTTCTTTGACCCCGATGCTAAA<br>GGCCTGAGCGACCCTGGCAAGATCAAGCGGCTGAGGAGCCAGGTGCAGGTCTCCCTGGAAGACTACATCAACGAT<br>CGCCAGTATGACTCCCGCGGACGATTTGGCGAGCTGCTGCTGCTGCTGCCTACTCTGCAGTCTATTACCTGGCAG<br>ATGATCGAACAGATTCAGTTCATCAAGCTGTTTGGCATGGCCAAAATCGATAATCTGCTGCAGGAGATGCTGCTG<br>GGAGGAAGTCCATCAGACGCTCCTCACGCACACCATCCACTGCACCCCCATCTGATGCAGGAGCATATGGGGACT<br>AACGTGATCGTCGCTAATACTATGCCCACCCACCTGAGTAACGGGCAGATGTGCGAATGGCCACGACCAAGAGGA<br>CAGGCAGCTACACCTGAGACTCCTCAGCCATCCCCACCTGGAGGAAGCGGATCCGAACCATACAAGCTGCTGCCC<br>GGCGCTGTGGCAACAATTGTCAAACCCCTGTCCGCCATTCCCCAGCCTACCATCACAAAGCAGGAAGTGATCTAG
TERT	ATGCCAAGAGCACCAAGATGCCGAGCCGTGAGAAGTCTGCTGAGATCCCATTATAGAGAGGTCCTGCCCCTGGCT<br>ACATTCGTGAGACGCCTGGGCCCACAGGGGTGGCGACTGGTCCAGCGAGGGGACCCAGCAGCTTTTCGGGCACTG<br>GTGGCACAGTGCCTGGTGTGCGTGCCATGGGATGCACGACCACCTCCAGCAGCACCTAGTTTCAGACAGGTGTCA<br>TGCCTGAAAGAACTGGTCGCACGGGTGCTGCAGCGGCTGTGCGAGAGGGGAGCCAAGAACGTGCTGGCCTTCGGA<br>TTTGCTCTGCTGGACGGAGCAAGGGGAGGACCACCTGAGGCCTTTACCACAAGTGTCCGCTCATACCTGCCAAAT<br>ACCGTGACAGATGCACTGCGAGGATCCGGAGCATGGGGACTGCTGCTGCGAAGAGTGGGAGACGATGTGCTGGTC<br>CACCTGCTGGCTAGATGCGCACTGTTCGTGCTGGTCGCACCCTCTTGCGCATACCAGGTGTGCGGACCACCACTG<br>TATCAGCTGGGAGCTGCAACCCAGGCTAGGCCTCCACCACACGCAAGCGGACCAAGGCGCCGACTGGGATGTGAA<br>CGGGCTTGGAACCATTCCGTCAGAGAGGCAGGAGTGCCTCTGGGACTGCCAGCACCTGGGGCTCGGAGAAGGGGA<br>GGCAGTGCCTCAAGGAGCCTGCCACTGCCAAAGCGACCACGACGAGGAGCAGCTCCTGAACCAGAGAGAACTCCA<br>GTGGGACAGGGCAGCTGGGCACACCCAGGAAGGACCCGAGGACCTTCAGACAGGGGATTCTGCGTGGTCAGCCCT<br>GCACGACCAGCAGAGGAAGCTACAAGCCTGGAGGGGGCTCTGTCCGGAACTCGACACAGCCATCCATCCGTGGGC<br>CGCCAGCACCATGCAGGACCTCCATCCACCTCTCGACCACCTCGGCCTTGGGACACACCATGTCCACCCGTGTAC<br>GCCGAAACTAAACACTTTCTGTATAGCTCCGGAGATAAGGAGCAGCTGCGGCCAAGTTTCCTGCTGTCTAGTCTG<br>AGACCCTCACTGACTGGCGCCCGGAGACTGGTGGAAACCATCTTTCTGGGAAGCAGACCCTGGATGCCTGGAACA<br>CCAAGGCGCCTGCCACGACTGCCTCAGCGATACTGGCAGATGAGGCCCCTGTTCCTGGAGCTGCTGGGGAATCAC<br>GCCCAGTGCCCTTATGGAGTGCTGCTGAAAACCCATTGTCCCCTGAGAGCAGCAGTCACACCTGCTGCAGGCGTG<br>TGCGCAAGGGAAAAGCCACAGGGAAGCGTGGCAGCTCCAGAGGAAGAGGACACCGATCCTCGACGGCTGGTGCAG<br>CTGCTGCGACAGCACTCAAGCCCCTGGCAGGTCTACGGATTCGTGCGGGCCTGTCTGAGAAGGCTGGTGCCTCCA<br>GGACTGTGGGGAAGCCGGCACAACGAACGCCGATTTCTGAGAAACACTAAGAAATTCATCTCCCTGGGCAAGCAT<br>GCTAAACTGTCTCTGCAGGAGCTGACCTGGAAGATGTCCGTGAGGGACTGCGCATGGCTGCGGAGATCTCCAGGA<br>GTCGGATGCGTGCCAGCAGCAGAGCACCGACTGCGAGAAGAGATTCTGGCCAAGTTTCTGCATTGGCTGATGAGC<br>GTGTACGTGGTCGAACTGCTGCGCTCCTTCTTTTATGTGACAGAGACTACCTTCCAGAAAAACAGACTGTTCTTT<br>TATAGGAAGAGCGTGTGGAGTAAACTGCAGTCTATCGGCATTCGGCAGCACCTGAAGCGGGTCCAGCTGAGAGAA<br>CTGAGTGAAGCCGAGGTGAGACAGCATAGGGAGGCACGACCTGCCCTGCTGACCAGCAGACTGAGGTTCATCCCC<br>AAGCCTGACGGACTGCGCCCAATTGTGAACATGGATTACGTGGTCGGAGCACGGACCTTCCGGCGAGAAAAACGA<br>GCTGAGCGGCTGACTTCTAGAGTCAAGGCACTGTTCAGTGTGCTGAATTATGAGAGGGCACGACGGCCCGGACTG<br>CTGGGAGCTTCCGTGCTGGGCCTGGACGATATCCACAGAGCCTGGAGGACCTTTGTCCTGCGCGTGCGAGCTCAG<br>GACCCACCTCCAGAACTGTACTTCGTGAAGGTCGATGTGACTGGGGCATATGACACCATCCCACAGGATAGACTG<br>ACCGAAGTGATCGCCTCTATCATCAAGCCCCAGAATACATACTGCGTGAGAAGGTATGCCGTGGTCCAGAAGGCT<br>GCACACGGCCATGTCAGGAAGGCTTTTAAATCACACGTGAGCACTCTGACCGACCTGCAGCCTTACATGAGACAG<br>TTCGTGGCTCATCTGCAGGAGACATCCCCACTGAGGGACGCAGTGGTCATCGAACAGTCCTCTAGTCTGAACGAG<br>GCTTCAAGCGGCCTGTTCGACGTGTTCCTGAGATTCATGTGCCACCATGCAGTCCGGATTAGAGGGAAAAGCTAC<br>GTGCAGTGTCAGGGCATCCCTCAGGGGTCCATTCTGTCTACCCTGCTGTGCAGCCTGTGCTATGGCGACATGGAA<br>AATAAGCTGTTTGCTGGGATCCGCCGAGATGGACTGCTGCTGCGCCTGGTCGACGATTTTCTGCTGGTGACACCC<br>CACCTGACTCATGCCAAAACCTTCCTGAGGACACTGGTCCGCGGAGTGCCAGAGTACGGATGCGTGGTCAACCTG<br>AGAAAGACAGTGGTCAATTTCCCTGTCGAAGATGAGGCTCTGGGGGGAACTGCATTTGTGCAGATGCCTGCCCAC<br>GGACTGTTCCCATGGTGTGGCCTGCTGCTGGACACAAGGACTCTGGAGGTGCAGTCTGATTACTCCTCTTATGCC<br>CGAACCAGTATTCGGGCTTCACTGACCTTCAACCGCGGATTCAAGGCCGGCCGAAATATGCGGAGAAAACTGTTT<br>GGCGTGCTGCGGCTGAAGTGCCATTCTCTGTTCCTGGACCTGCAGGTCAACAGTCTGCAGACCGTGTGCACAAAT<br>ATCTACAAAATTCTGCTGCTGCAGGCCTATCGCTTTCACGCTTGCGTCCTGCAGCTGCCTTTCCATCAGCAAGTG<br>TGGAAGAACCCAACTTTCTTTCTGAGAGTGATCAGCGATACCGCTTCCCTGTGCTACTCTATTCTGAAGGCTAAA<br>AATGCAGGCATGAGTCTGGGAGCAAAAGGAGCAGCTGGACCACTGCCCTCAGAAGCAGTGCAGTGGCTGTGCCAC<br>CAGGCCTTTCTGCTGAAGCTGACAAGGCATCGCGTCACTTATGTGCCACTGCTGGGGTCACTGAGGACTGCCCAG<br>ACCCAGCTGAGCCGCAAACTGCCCGGAACAACTCTGACCGCTCTGGAGGCAGCCGCTAACCCAGCACTGCCCAGC<br>GACTTCAAGACAATCCTGGATTAG

CTNNB1	ATGGCAACACAGGCCGACCTGATGGAACTGGATATGGCAATGGAACCCGACAGAAAGGCAGCCGTCTCACACTGG<br>CAGCAGCAGAGCTACCTGGATTCCGGGATCCACTCTGGAGCAACCGCCACAGCTCCCTCACTGAGCGGGAAGGGA<br>AACCCTGAGGAAGAGGACGTCGATACCAGCCAGGTGCTGTACGAATGGGAGCAGGGCTTCTCCCAGTCTTTTACA<br>CAGGAGCAGGTGGCCGACATCGATGGGCAGTATGCAATGACACGAGCTCAGAGGGTGCGCGCAGCTATGTTCCCC<br>GAAACTCTGGATGAGGGCATGCAGATTCCTTCCACACAGTTTGACGCAGCCCACCCAACTAATGTGCAGAGGCTG<br>GCCGAACCCTCTCAGATGCTGAAGCATGCTGTGGTCAACCTGATCAATTATCAGGACGATGCTGAACTGGCAACT<br>CGCGCCATTCCAGAGCTGACCAAGCTGCTGAACGACGAAGATCAGGTGGTCGTGAACAAGGCTGCAGTGATGGTC<br>CACCAGCTGAGCAAGAAAGAGGCATCCCGACATGCCATCATGCGGTCTCCCCAGATGGTCAGTGCCATTGTGAGG<br>ACCATGCAGAACACAAATGATGTGGAGACAGCTCGCTGTACAGCAGGCACTCTGCACAACCTGAGTCACCATAGG<br>GAAGGGCTGCTGGCAATCTTCAAGTCAGGCGGGATTCCTGCCCTGGTGAAAATGCTGGGCAGTCCAGTGGACTCA<br>GTCCTGTTTTACGCCATCACCACACTGCACAATCTGCTGCTGCATCAGGAGGGGGCTAAGATGGCAGTGCGCCTG<br>GCAGGAGGCCTGCAGAAAATGGTCGCCCTGCTGAACAAGACCAATGTGAAATTCCTGGCCATCACTACCGACTGC<br>CTGCAGATTCTGGCTTATGGCAACCAGGAGTCAAAGCTGATCATTCTGGCAAGCGGAGGACCACAGGCTCTGGTG<br>AATATCATGCGAACTTACACCTATGAAAAACTGCTGTGGACAACTTCCAGAGTGCTGAAGGTCCTGTCTGTGTGT<br>AGCTCCAACAAACCTGCCATTGTGGAGGCTGGAGGAATGCAGGCACTGGGACTGCACCTGACAGATCCATCACAG<br>CGCCTGGTGCAGAACTGCCTGTGGACCCTGCGAAATCTGAGCGACGCCGCTACAAAGCAGGAAGGCATGGAGGGG<br>CTGCTGGGAACTCTGGTGCAGCTGCTGGGGTCTGACGATATCAATGTCGTGACTTGCGCAGCCGGAATTCTGAGT<br>AACCTGACCTGTAACAATTACAAGAACAAGATGATGGTCTGCCAAGTGGGAGGCATCGAGGCTCTGGTCCGAACA<br>GTGCTGCGGGCAGGGGACAGAGAAGATATCACTGAGCCTGCCATTTGTGCTCTGAGACACCTGACCAGTAGGCAT<br>CAGGAAGCAGAGATGGCCCAGAACGCTGTGCGGCTGCACTATGGCCTGCCAGTCGTGGTCAAGCTGCTGCACCCC<br>CCTTCTCATTGGCCCCTGATCAAAGCTACCGTGGGGCTGATTAGAAACCTGGCACTGTGCCCTGCAAATCACGCA<br>CCACTGCGAGAGCAGGGAGCAATCCCACGACTGGTCCAGCTGCTGGTGCGGGCTCATCAGGATACTCAGCGGAGA<br>ACCTCCATGGGGGGAACTCAGCAGCAGTTCGTCGAGGGCGTGCGGATGGAAGAGATCGTGGAAGGCTGTACCGGG<br>GCACTGCACATTCTGGCCAGAGACGTCCATAACAGGATCGTGATTCGCGGACTGAATACCATCCCCCTGTTTGTG<br>CAGCTGCTGTACAGCCCTATCGAGAACATTCAGAGAGTCGCTGCAGGCGTGCTGTGCGAACTGGCCCAGGATAAG<br>GAAGCCGCTGAGGCTATTGAAGCAGAGGGAGCAACAGCCCCCCTGACTGAACTGCTGCACAGCAGGAATGAGGGC<br>GTCGCCACATACGCAGCCGCTGTGCTGTTCCGCATGAGCGAGGACAAACCCCAGGATTATAAGAAACGGCTGTCC<br>GTGGAACTGACCTCTAGTCTGTTTAGAACAGAACCTATGGCTTGGAACGAGACCGCAGACCTGGGACTGGATATC<br>GGAGCACAGGGAGAGCCACTGGGATACAGACAGGACGATCCCAGTTATAGGTCATTCCACAGCGGAGGATACGGA<br>CAGGATGCACTGGGCATGGACCCTATGATGGAACATGAGATGGGAGGACACCATCCAGGAGCAGACTATCCTGTG<br>GATGGGCTGCCAGACCTGGGACATGCTCAGGACCTGATGGATGGCCTGCCACCCGGGGACAGCAATCAGCTGGCC<br>TGGTTTGACACCGATCTGTAG
